# A new and cryptic species of *Lissodesmus* Chamberlin, 1920 (Diplopoda, Polydesmida, Dalodesmidae) from Tasmania, Australia

**DOI:** 10.3897/zookeys.846.35028

**Published:** 2019-05-16

**Authors:** Robert Mesibov

**Affiliations:** 1 West Ulverstone, Tasmania 7315, Australia Unaffiliated West Ulverstone Australia

**Keywords:** Australia, Dalodesmidae, Diplopoda, Polydesmida, Tasmania

## Abstract

*Lissodesmuspiscator***sp. nov.** differs from the 30 previously described *Lissodesmus* species in the form of the femoral process of the gonopod telopodite, which is tripartite with an erect distal branch and two posteromedially curving basal branches. Despite careful searching, the new species has only been collected by pitfall trapping and may have a very small range in the northwest corner of the Central Plateau in Tasmania, Australia.

## Introduction

**Figure 1.**
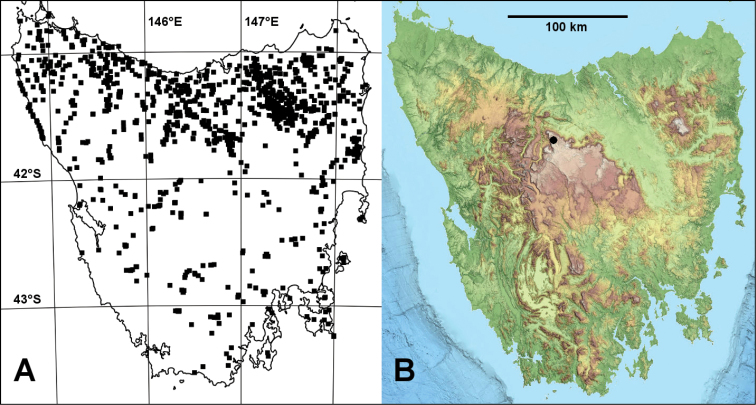
*Lissodesmus* localities in Tasmania (excluding Bass Strait islands) **A** localities for *Lissodesmus* specimens identified to species (black squares) from *Atlas of Living Australia* (https://collections.ala.org.au/public/show/dr444, accessed 10 January 2019) **B** locality for *L.piscator* sp. nov. (black circle) on coloured relief map from *theLIST* (https://maps.thelist.tas.gov.au/listmap/app/list/map) State of Tasmania. Mercator projections.

The genus *Lissodesmus* Chamberlin, 1920 currently includes 19 species in Tasmania and 11 species in Victoria; see Mesibov (2018) for a genus synonymy and list of species. Although the Tasmanian *Lissodesmus* fauna has been well sampled over many years (Fig. 1A), a narrow-range alpine species was only first collected in 2017 (Mesibov 2018) and additional narrow-range species probably remain to be discovered.

The new *Lissodesmus* species described in this paper might represent another category of undescribed Tasmanian millipedes: possibly more widely distributed, but unusually cryptic. It was pitfall-trapped at a single location in the summers of 2017, 2018 and 2019, but has not yet been collected by hand in the pitfall area, despite careful searching.

## Materials and methods

### Pitfall trapping

Pitfall traps were set and emptied by Michael Driessen of the Department of Primary Industries, Parks, Water and Environment (DPIPWE), Tasmania. The trapping was part of a wildfire recovery study following a fire that burned from January to March 2016 and covered ca. 26,000 ha south and west of Lake Mackenzie, a hydroelectric impoundment at the northwest corner of Tasmania’s Central Plateau (Natural Values Conservation Branch 2017). Post-fire monitoring studies of flora and fauna were initiated shortly after the fire had been brought under control.

**Figure 2.**
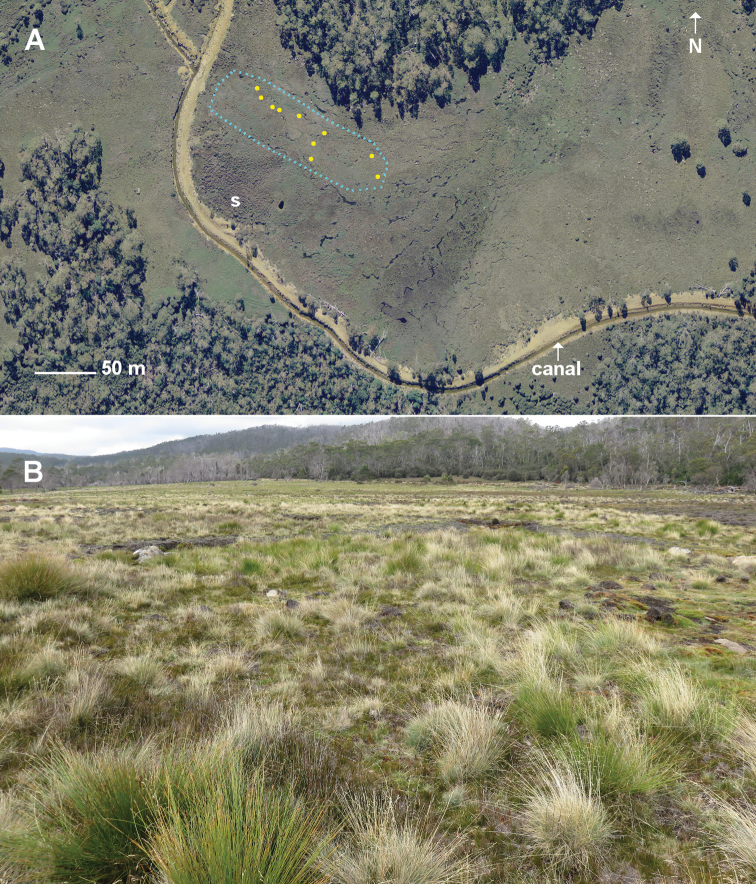
Ritters Plain pitfall area and surrounds **A** pre-fire aerial photo (14 April 2014) showing the pitfall area (blue dotted line), locations of pitfall traps with *Lissodesmuspiscator* sp. nov. (yellow dots), a dense shrubbery of *Richeascoparia* (**s**) and the canal draining this portion of the Plain **B** view to the southeast on 28 March 2019 from a point near the western end of the pitfall area.

The pitfall traps were set on Ritters Plain (Fig. 2), ca. 2.5 km west of Lake Mackenzie and ca. 1 km south of the Fisher River gorge. Each trap was a 225 ml plastic cup placed with its rim flush with the ground surface in a short section of 75 mm PVC pipe fitted snugly in a hole drilled with an auger. The traps were placed in each of three burn categories: unburned vegetation and unburned peat, burned vegetation and unburned peat (lightly burned) and burned vegetation and burned peat (deeply burned). Ten traps were irregularly placed a minimum of 5 m apart in each category, reflecting the patchy nature of the burn. Each trap cup was filled with 100 ml of 70% ethanol over a few ml of glycerol, covered with a rain shield and left for 14 days in the late austral summer: 20 February – 6 March 2017, 22 February – 8 March 2018 and 20 February – 6 March 2019.

Millipedes and other invertebrates in the traps were sorted for DPIPWE by Kevin Bonham, who sent specimens of an unfamiliar *Lissodesmus* to the author for identification in May 2018.

### Millipede searches

I searched the Ritters Plain pitfall area and the nearby grassy sedgeland and subalpine forest unsuccessfully for fresh material of the new *Lissodesmus* on 8 December 2018 and on 20 February, 11 March and 28 March 2019, for a total of ca. 8.5 hours over ca. 20 ha. I collected representative specimens of other millipede species on each visit. Microhabitats examined included woody litter, bark litter, leaf litter, grass and sedge turf, and the spaces under stones and under prostrate (rock-hugging) shrubs. I also excavated small deposits of peaty soil underlying elevated *Sphagnum* moss mounds on the south side of the Plain.

### Specimen preparation

All specimens of the new *Lissodesmus* species are stored in 80% ethanol in the Queen Victoria Museum and Art Gallery. The paratype male was briefly treated with vinegar to reduce its stiffness. Specimens were examined and measured using a Nikon SMZ800 binocular dissecting microscope. Focus-stacks of colour images were manually generated using a Canon EOS 1000D digital SLR camera mounted on the Nikon SMZ800 fitted with a beam splitter, then processed with Zerene Stacker 1.04 software. The gonopods of a male from 2018 pitfall trapping were cleared in 80% lactic acid, temporarily mounted in a 1:1 glycerol:water mixture and imaged using an eyepiece video camera mounted on an Amscope binocular microscope. Preliminary drawings were traced from printed copies of the images, then corrected by reference to the actual gonopods. Figures were composed using GIMP 2.8 and the map in Fig. 1A with QGIS 2.14.

Specimen locality data are provided in Supplement material 1 in Darwin Core format. Pitfall trap locations were provided by Michael Driessen as a site map based on a georegistered aerial photograph (see Fig. 2A). Locations of the 10 traps yielding the new *Lissodesmus* species have been spatially summarised as -41.6824 146.3524 ±75 m (WGS84 datum).

The terminology of gonopod telopodite parts follows Mesibov (2006).

Repositories, institutional acronyms, or institutional abbreviations: **QVMAG**, Queen Victoria Museum and Art Gallery, Launceston, Tasmania.

## Results

### Pitfall trapping

Twelve specimens of a new *Lissodesmus* species (described below) were found in five 2017 traps, five 2018 traps, and one 2019 trap (Fig. 2A). Four of the traps were in lightly burned ground and six in unburned ground; one unburned-ground trap location had a single specimen in each of 2017 and 2018. In three trapping seasons, the traps also yielded eight specimens of *Paredrodesmusmonticolus* Mesibov, 2003 (Polydesmida, suborder Dalodesmidea), two of *Australeumasimile* Golovatch, 1986 (Chordeumatida, Metopidiotrichidae), and one each of *Amastigogonusfossuliger* Verhoeff, 1944 (Spirostreptida, Iulomorphidae) and *Atrophotergummontanum* Mesibov, 2004 (Polydesmida, Dalodesmidae).

### Millipede searches

As mentioned in the Materials and methods section, I found no *Lissodesmus* specimens in or near the pitfall area. However, I had little difficulty finding the four other trapped species, as well as four more: *Gasterogrammapsi* Jeekel, 1982 and “M20” (both Polydesmida: Dalodesmidae, the latter undescribed but recorded elsewhere in northwest Tasmania), an unidentified *Procyliosoma* sp. (Sphaerotheriida, Procyliosomatidae) and the undescribed but well-recorded siphonotid “AcuMes” (Polyzoniida: Siphonotidae).

### Order Polydesmida Pocock, 1887

#### Suborder Dalodesmidea Hoffman, 1980

##### Family Dalodesmidae Cook, 1896

###### Lissodesmus piscator sp. nov.

Taxon classificationAnimaliaPolydesmidaDalodesmidae

http://zoobank.org/50E0322D-8436-4443-A613-8E644A695995

Figs 3 , 4

####### Holotype.

AUSTRALIA • male; Tasmania, Central Plateau, Ritters Plain; [41.6824°S 146.3524°E]; 1080 m a.s.l.; 6 Mar. 2019; M. Driessen leg.; pitfall PU9 20 Feb.–6 Mar. 2019; coordinates are center of cluster of pitfall traps yielding *L.piscator* sp. nov. in 2017, 2018, 2019; coordinate uncertainty 75 m; QVMAG: QVM:2019:23:0015.

####### Paratype.

AUSTRALIA • male; same data as for holotype; dissected; QVMAG: QVM:2019:23:0016.

####### Other material.

5 males, 4 females and 1 stadium 7 female, same locality as holotype; see Supplement material 1 for details.

####### Diagnosis.

Distinguished from all other known *Lissodesmus* species by the form of the femoral process on the gonopod telopodite: the process has an erect, flattened, bluntly toothed distal branch and two large basal branches curving posteromedially across the posterior face of the telopodite.

####### Description.

**Figure 3.**
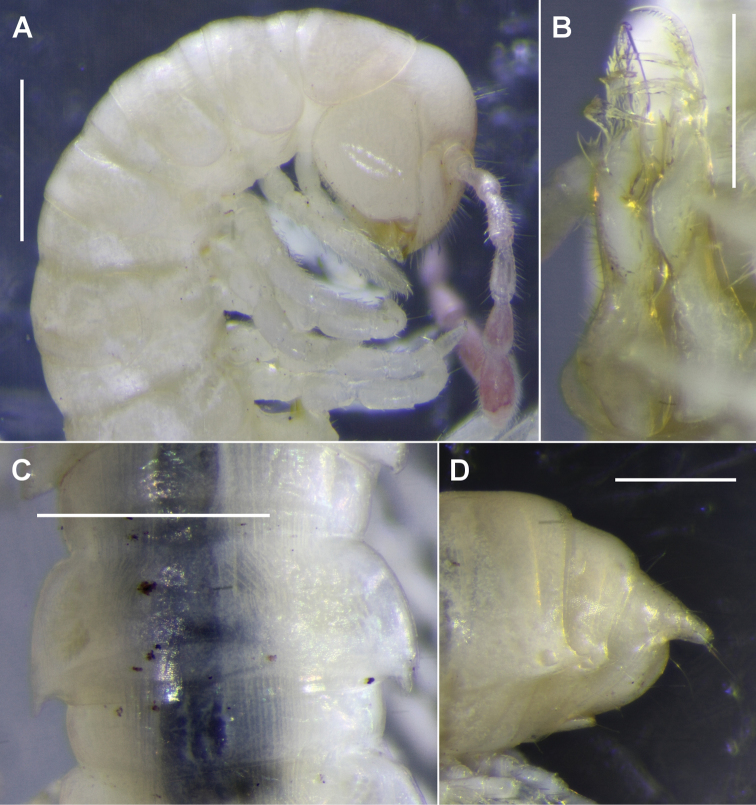
*Lissodesmuspiscator* sp. nov., holotype (**A, D**) and paratype (**B, C**) **A** right lateral view of head and anterior rings **B** ventral and slightly left-lateral view of gonopods in situ **C** dorsal view of midbody rings **D** left lateral view of telson. Scale bars 1.0 mm (**A, C**); 0.5 mm (**B, D**).

Male/female approximate measurements: length 15/20 mm, midbody vertical diameter 1.3/1.6 mm, midbody width across paranota 1.7/1.7 mm. Colour in alcohol almost uniformly pale, antennae roseate (Fig. 3A).

Male with clypeus and frons moderately setose, vertex sparsely setose. Antennal sockets separated by ca. 2.5X socket diameter. Antenna short, just reaching ring 3 when manipulated backwards; relative length of antennomeres 6 > (2,3) > (4,5), antennomere 6 widest. Head approx. as wide as tergite 4, cardines in dorsal view quadrate in outline; collum narrower than head and tergite 2; anterior collum margin gently convex, curvature extending smoothly to slightly convex lateral margin; posterior margin more or less straight; corners bluntly pointed. Tergite width increasing gradually from rings 2–6, then subequal, then decreasing 17–19. Waist pronounced (Fig. 3C), with faint longitudinal striations. Prozonites and metazonites with faint cellular sculpturing; limbus composed of narrow, distally tapered tabs. Paranota (Fig. 3C) smooth, narrow (ratio of overall width to prozonite width ca. 1.2 on midbody ring); anterior shoulder gently curving into slightly convex lateral margin, the latter with a small notch posteriorly and with short seta on anterior corner of notch; posterior corner upturned, extending just past posterior metatergite margin on most rings, usually with small subterminal seta; posterior corner seta prominent, erect (broken off on some rings). Ozopore small, round, opening dorsally close to paranotal margin and anterior to posterior corner; pore formula 5, 7, 9, 10, 12, 13, 15–19. Spiracles on diplosegments small, round, not emergent; rims slightly raised above pleural surface; posterior spiracle approx. midway between leg bases. Sternites approx. as long as wide, with short, sparse setae; transverse impression more pronounced than longitudinal. Legs short, ca. 0.9x as long as maximum ring diameter at midbody; legpairs 3–9 with prefemur slightly swollen dorsally; relative podomere lengths tarsus > (femur, prefemur) > (postfemur, tibia) on midbody legs; tarsus straight, ca. 1.3x as long as femur. From legpair 3, sphaerotrichomes on femur, postfemur, tibia and tarsus, densest on tibia and tarsus; sphaerotrichome numbers rapidly diminishing posteriorly but with a few sphaerotrichomes on tibia and tarsus of posterior legs; no sphaerotrichomes on last two legpairs; each sphaerotrichome hemispherical with tapering, blunt-tipped seta inclined distoventrally. Dense brush setae with slightly expanded tips on prefemur and femur of anterior legs; brush setae disappearing posteriorly. Pre-anal ring (Fig. 3D) with sparse, long setae; hypoproct trapezoidal, dorsal margin straight; epiproct extending past anal valves, tapering to truncate tip ca. 1/10 maximum width of ring 19; spinnerets in square array in shallow cavity just ventral to epiproct tip.

Gonopore small, opening mediodistally on only slightly enlarged leg 2 coxa. Bases of legs 6 and 7 well-separated by shallowly concave sternite, bases of legs 5 closer; brushes of sparse, long setae on sternites just medial to coxae of legs 5, 6, 7. Aperture ovoid, wider than long, ca. 1/2 width of ring 7 prozonite, rim slightly raised laterally and posteriorly.

**Figure 4.**
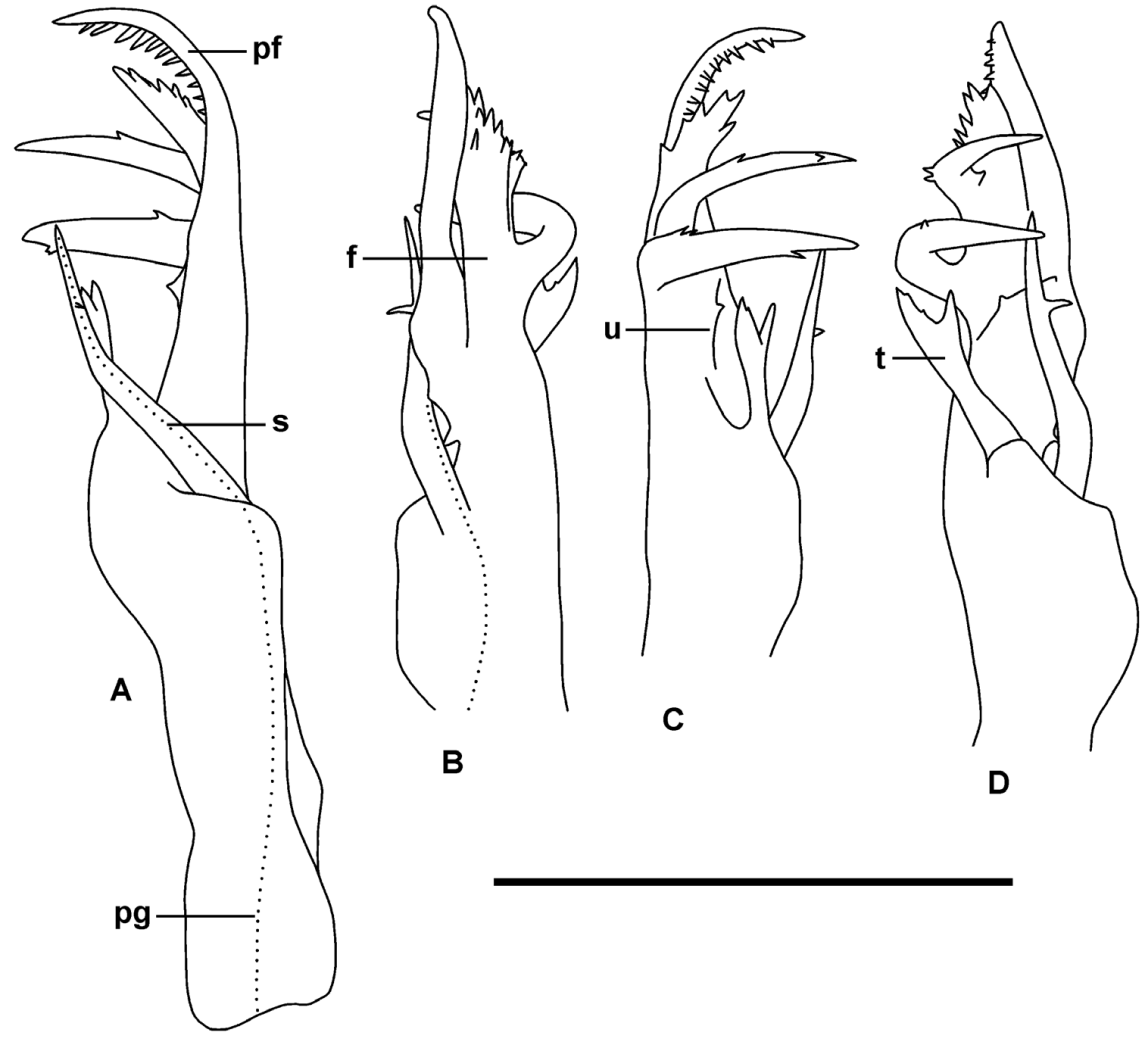
*Lissodesmuspiscator* sp. nov., QVM:2018:23:0053. Right gonopod telopodite in medial (**A**) anterior (**B**) lateral (**C**) and posterior (**D**) views. Abbreviations: f = femoral process, pf = prefemoral process, pg (dotted line) = prostatic groove, s = solenomere, t = tibiotarsus, u = uncus. Setation not shown. Scale bar 0.5 mm.

Gonopods: Gonocoxae truncate-conical, lightly joined distomedially. Telopodite (Figs 3B, 4) slender, erect, extending to leg 5 bases when retracted; moderately setose on posterolateral surface from base to level of tibiotarsus origin. Solenomere (Fig. 4, s) slender, tapering, arising anteromedially at 1/3-1/2 telopodite height and terminating at ca. 3/4 telopodite height; directed posterodistally, slightly bent laterally at ca. 2/3 solenomere height, with small, medially directed, subapical tooth. Prostatic groove (Fig. 4, pg) running on anteromedial surface of telopodite to solenomere base, opening at solenomere tip. Tibiotarsus (Fig. 4, t) arising at ca. 1/2 telopodite height in large, anteromedial flange on telopodite; directed distolaterally above thickening on telopodite surface, slightly flattened anteroposteriorly, terminating in wide “Y” at level of subapical tooth on solenomere. Prefemoral process (Fig. 4, pf) tapering distally to bluntly rounded, posteriorly curved tip; subapically with posteroventral comb of 10-12 strong, well-spaced teeth; uncus (Fig. 4, u) at ca. 1/2 prefemoral process height, oblique, with small medial tooth; a small, sharp tooth on mediobasal surface of prefemoral process. Femoral process (Fig. 4, f) arising on anterolateral surface of prefemoral process, closely appressed to distal portion of latter; divided into erect distal branch, slightly expanded distally with small, blunt, marginal and submarginal teeth, the branch terminating just below level of prefemoral process tip; and 2 stout, well-separated basal branches curving posteriorly and medially, the most basal branch reaching and almost touching the solenomere tip, both branches with a few small teeth.

Female closely resembling male but stouter. Genital aperture with posterior margin gently convex medially; cyphopods not examined.

####### Name.

Latin *piscator*, fisherman, noun in apposition, for the type locality in the Fisher River catchment.

####### Distribution.

So far known only from Ritters Plain near Lake Mackenzie in northwest Tasmania (Figs 1B, 2). The Plain has a habitat area of ca. 100 ha and a known occupied area of less than 1 ha.

## Discussion

### In what microhabitats are *L.piscator* sp. nov. living?

Ritters Plain is almost entirely treeless (Fig. 2). Most of the Plain is covered by a tightly intergrown turf of grass and sedge species overlying a dense, fibrous, anaerobic peat, mixed with patches of slightly elevated *Sphagnum* bog (Department of Primary Industries, Parks, Water & Environment 2016, Natural Values Conservation Branch 2017). Annual rainfall at nearby Lake Mackenzie is ca. 2100 mm and snow lies on the Plain during the winter months. For much of the year the soil is saturated with water. The pitfall area was probably even wetter before a canal (Fig. 2A) was constructed in the 1960s on the uphill side of the Plain to capture additional water for the Fisher River hydroelectric project.

There are no above-ground shelters near the pitfall traps in which *L.piscator* sp. nov. was found, so the population is probably living underground, either in crevices in the root-filled turf or in cavities among the periglacially shattered rock fragments that cover much of this portion of the Central Plateau and underlie the peaty soil. Adults might be expected to come to the surface to mate and disperse during late summer — when the pitfall trapping was carried out — and 11 of the 12 specimens trapped were adults.

The densest populations of millipedes I found by searching on Ritters Plain were in surface peat associated with elevated *Sphagnum* moss mounds, such as the ones surrounding the lower parts of *Richeascoparia* stems just to the southwest of the pitfall area (s in Fig. 2A). The foliage and fine branches of the *Richea* were burned in the 2016 fire, but many of the lower stems buried in moss were still alive in 2019. The moss mounds themselves, with and without *Richea*, were largely unburned. In the peat close to the moss I frequently found *G.psi* and *Procyliosoma* sp., and occasionally small groups of *P.monticolus* and *A.fossuliger*. I was surprised not to find *L.piscator* sp. nov. in the peat as well, since *Lissodesmus* spp. cohabit with *Gasterogramma* spp. elsewhere in Tasmania.

### Does *L.piscator* sp. nov. have a very narrow range, or is it unusually cryptic, or both?

I collected repeatedly above ca. 1000 m in the Lake Mackenzie area in the period 1985-2007, finding the polydesmidans *A.montanum*, *G.psi*, *P.monticolus* and *Bromodesmusrufus* Mesibov, 2004 (Dalodesmidae), but no *Lissodesmus* species. The only previous *Lissodesmus* records from the catchments of the Fisher River and its tributary the Little Fisher River are for the common northwest Tasmanian species *L.perporosus* Jeekel, 1984 at somewhat lower elevations (to 920 m; records in *Atlas of Living Australia*, https://collections.ala.org.au/public/show/dr444; accessed 10 January 2019). The nearest high-elevation *L.perporosus* locality is ca. 12 km to the east of Ritters Plain, at ca. 1150 m (higher than Ritters Plain) in the headwaters of Western Creek.

If *L.piscator* sp. nov. is largely a subterranean species, the fact that it has not yet been hand-collected is not surprising. It is unlikely that it only occurs in the small portion of Ritters Plain that coincidentally was sampled with pitfalls in the post-fire recovery study. I suspect that it also occurs nearby in the voids in the periglacial scree deposits that cover slopes with boulder-sized rocks in the northwest corner of the Central Plateau. Sampling in the screes would be even more difficult than setting pitfall traps over a wide area, and the true distribution and conservation status of *L.piscator* sp. nov. are likely to remain indeterminate.

## Supplementary Material

XML Treatment for Lissodesmus piscator

## Supplementary materials

Supplementary material 1 Specimen data for *Lissodesmuspiscator* sp. nov. Data type: occurrence Explanation note: Data file Specimen_data_Lissodesmus_piscator_2019.tsv for 12 specimen lots of Lissodesmuspiscator sp. nov. in the Queen Victoria Museum and Art Gallery, Launceston, Tasmania, Australia. The file is a tab-separated table in UTF-8 encoding with the following Darwin Core fields: institutionCode, catalogNumber, phylum, class, order, family, genus, specificEpithet, scientificName, typeStatus, organismRemarks, locality, country, stateProvince, decimalLatitude, decimalLongitude, geodeticDatum, coordinateUncertaintyInMeters, georeferenceSources, georeferencedBy, georeferenceRemarks, minimumElevationInMeters, recordedBy, eventRemarks and eventDate. This dataset is made available under the Open Database License (http://opendatacommons.org/licenses/odbl/1.0/). The Open Database License (ODbL) is a license agreement intended to allow users to freely share, modify, and use this Dataset while maintaining this same freedom for others, provided that the original source and author(s) are credited.Robert Mesibov
